# Elements of Pathological Anatomy, Illustrated by Numerous Engravings

**Published:** 1840-08

**Authors:** 


Art. VI.—*Elements of Pathological Anatomy, illustrated by numerous engravings.* "In Morbis, sive acutis, sive chronicis, viget occultum, per humanas speculationes incomprehensible." Baglivi. By Samuel D. Gross, M.D. Late professor of General Anatomy, Physiology, and Pathological Anatomy, in the Medical Department of the Cincinnati College. Vol. II, 8vo., Boston, 1839. Marsh, Capen, Lyon & Webb and James B. Dow.

Many of our brethren must well remember the time, when the only work on Morbid Anatomy within their reach, was the little volume of 250 pages, from the pen of Dr. Matthew Baillie of London, re-printed in Albany in 1795. It was altogether *special.* Since that date, such has been the progress of this new department, that the *general* has been added to the special—systems have been formed, and a new branch of science created. Previously, however, to the re-publication in this country, of Andral's celebrated "Treatise on Pathological Anatomy," eight years ago, the American student had but meagre opportunities for the prosecution of this most interesting and useful study. The advancement which has been made since that work appeared, may be inferred from the fact, that several medical schools have been enriched with professorships of Morbid Anatomy, and, still more, from the fact now before us, that a physician of our own country—a practitioner of the west—has brought out a well-digested work of equal extent, and greater simplicity than Andral's, which, we learn, is every where well received by the profession.

The eminent French pathologist attempted to present the facts of this science, by a method that would disclose the origin of the morbid appearances which he described. Thus he formed the great classes—lesions of circulation—of nutrition, of secretion, of innervation, and of the blood. It is certainly advantageous to the student to contemplate them by the lights of this system, but we cannot admit that it is without difficulties. Thus it rarely, if ever, happens, that one of the great functions can be disordered, and the others remain unaffected; and in the majority of cases the lesion of structure is manifestly the result of irregularity in more than one of the functions. To select a simple case, the hypertrophy of an organ, although, immediately, the effect of an excessive energy of the nutritive function, is generally attended equally with an excess in its circulation. But to choose a stronger and more complicated example—in induration what do we observe? A chronic inflammation, (lesion of the *circulation*,) sustained no doubt by a lesion of the *innervation*, a lesion of the *blood*, indicated by its siziness, and a lesion of *secretion*, if not indeed of *nutrition*. To refer an indurated tissue to any one of these heads is then, evidently, an arbitrary decision.

Perceiving this difficulty, professor Carswell of London, one of the ablest pathological anatomists of the age, in his splendid and expensive "Illustrations of the Elementary Forms of Disease," (he should have said, of *deranged structure*,) determined to class the morbid appearances according to their affinities. Thus, to cite a single example, he brings together all the cases of *softening*, however diverse the lesions of function generating them; and then proceeds to enumerate those lesions. This is the plan adopted by professor Gross, and we think it decidedly preferable to that of Andral.

Nevertheless, whatever method of classification the pathological anatomist may adopt, he must always commence with inflammation; for it is undeniable, that of all the modes of morbid action, this one is most prolific in those results, which are the immediate objects of his study.

To this great truth our author is quite alive. Indeed, he seems to favor the opinion, that *all* derangements of structure depend directly or indirectly on this kind of morbid action—more or less intense, and modified in its phenomena and effects, by the tissues in which it occurs, the nature of the remote cause, and the temperament of the patient. Thus he assigns the same proximate cause—inflammation, for the false membranes of the pleura, the tubercles of the lungs, the serum in hydropericardium, and scirrhous tumours—it being understood, however, that some of these inflammations are of a common kind—others specific. At the same time, he is a decided advocate for the doctrine, which teaches the local origin of *all* diseases, and the sympathetic origin of all constitutional affections. In this generalization, which reduces every morbid state to inflammation and constitutional disturbance, as its effect, there is certainly an attractive simplicity; but we are not yet fully prepared for its adoption. Used in this comprehensive sense, the word *inflammation* becomes nearly synonymous with the *morbid action* of our celebrated countryman Dr. Rush; which, in turn, is but an equivalent for disease—and these different forms of speech might indeed be indiscriminately employed.

A mode of morbid action is characterized, first, by the phenomena which it displays during its existence, second, by its effects. When both have been adequately observed, and are found to be constant, we admit, that occurrence of this mode in any particular case, may be established, either by the symptoms, or the morbid appearances, found after they have passed away. Whenever the well known phenomena of inflammation are present—or the appearances on examining a morbid structure are such as are known, at all times, to have been preceded by those phenomena, we must grant of course that the disease *is* inflammation. But we are not bound to grant, when neither the symptoms nor the morbid products *known* to result from inflammation show themselves, that the disease was inflammatory. Thus, if we observe swelling, redness, pain and heat, or a majority of these conditions, in a part, we say that it is inflamed; and if we find deposits of pus, or of coagulable lymph, in the form of false membranes, in tissues which have ceased to be the seats of morbid action, we admit their inflammatory origin, because observation has taught us, that inflammation and it *only* produces them. When, however, we find serum, scirrhus or tubercle, in parts which have not emitted the symptoms of inflammation, and unassociated with false membranes or purulent secretion, it is gratuitous to affirm, that they are the offspring of inflammation. The existence of any one of them in any particular case, cannot, therefore, be cited as evidence of previous inflammation, until it is shown by testimony, that inflammation has in other cases produced it.

We grant that these, and other morbid products of a like kind, *may* be the offspring of inflammation, and it is due to the class of pathologists of which our author is one of the ablest, to admit, that they are not without facts of a weighty character to sustain their conclusion.

*First.*—The vast number of cases in which inflammation is the undoubted cause of morbid structure, would seem on the principles of analogy, to warrant the conclusion, that *all* morbid structures are its consequence. This however is but presumptive evidence.

*Second.*—Many inflammations it must be confessed, from the nature of the tissues in which they are seated, and from their mildness, give little pain and awaken but few constitutional sympathies; they may, therefore, exist to the extent of producing deranged structure, without manifesting themselves by the usual signs; and it cannot be said, that certain serous effusions and non-analogous deposits, are not of this kind. Thus tubercle may be secreted under such an inflammatory action. But here again is nothing more than probability.

*Third.*—It is affirmed, that many inflammatory congestions disappear, when the patient is *in articulo mortis*, or immediately after death. When we recollect, however, that the difficulty of washing or expressing the blood, from an inflamed tissue, is given as an evidence of the existence of previous inflammation, we cannot grant, that the number of cases of *resolution* at the close of life is as great as some pathologists believe. We *conjecture*, moreover, that it is in cases of short duration rather than chronic, that the inflamed capillaries thus unburthen themselves.

*Fourth.*—Almost all deranged structures, manifestly depend on deposits from the blood. Now inflammation, in all its stages, is known to favor effusion, and it may, therefore, be presumed to occasion all that we find. It is, however, an unquestionable fact, that we *may* have copious and altered secretion without inflammation. Blood is extravasated into the cutaneous or subcutaneous parts in scorbutus—the kidneys, and intestinal mucous membrane, secrete copiously an altered fluid under certain mental emotions, and the skin pours out freely in the very hour of death. There is no inflammation in any of these cases.

*Fifth.*—It may be, that certain heterologous matters, such as tubercle, melanosis and scirrhus, take the place of the ordinary lymphatic deposits afforded by common inflammation, which latter do not therefore appear. This must be granted; but the possibility of such an operation cannot be admitted as evidence of its actual occurrence.

*Sixth.*—An argument on which our author lays great stress is, that the deposit of lymph is one of the most characteristic effects of inflammation, and that a part at least of the heterologous structures, when analysed, are found to be nearly of the same composition. Some of them, however, are highly albuminous. Moreover, all deposits must be from the blood, and all which solidify must be composed in a great degree of the coagulating or, at least, the coagulable parts of that fluid. The source and material, then, of the analogous and the non-analogous deposits, must be the same; but it by no means follows, that the *mode* of action which produced the extravasation is the same, and to it our attention is now directed.

*Seventh.*—The heterologous deposits possess in themselves the power of generating blood and vessels to circulate it, which inosculate with those of the tissue into which the deposit has been made; a phenomenon identical with what we see in false membranes. This fact is entitled to grave consideration; but we cannot admit its conclusiveness; for, in the first place, it has not yet been *established*, that all of these productions possess this self-organizing power; and secondly, if they do, it would not prove that the *action* which extravasated the altered lymph that generally enters into their composition, was inflammatory. We do not know, but that other modes of morbid action may throw out lymph capable of generating blood and vessels. When this is shown to be impracticable the controversy will be at an end.

*Lastly.*—All the non-analogous structures, are sooner or later associated with inflammation in the surrounding tissues, and most of them present it in their own substance. But this association, established long after they were deposited, can throw no light on the mode of morbid action which occasioned their deposit.

Our limits do not permit us to extend the inquiry into this subject, which, at last, is of more interest in a pathological, than a therapeutic view; for if most of the cases in dispute are of such a *specific* inflammatory nature, as to afford results so different from those of common inflammation, we cannot expect to cure them by simple antiphlogistics; and, indeed, experience has taught us, that the deposit of tubercle, scirrhus, encephaloid and melanosis, cannot be arrested by the means which subdue ordinary inflammation. It is, then, to their peculiar or specific, not their inflammatory or non-inflammatory character, that we should turn our attention.

Having indicated one of the great principles which pervade the first part of Professor Gross' book, we shall with greater conciseness refer to another. Although adopting the division of morbid structures into analogous and heterologous, he distinctly intimates that there is not much foundation in nature for this division. This in fact is but a corollary from the other proposition, to-wit, their derivation from a common source, through the same instrumentality, inflammation. But apart from the last of these considerations, we do not perceive much propriety in this distinction.

All of them are derived from the blood, by morbid secretion; they are all disposed to become vascular, and all bear a resemblance to the normal tissues, while none of them is perhaps identical with any one of those tissues, except the cellular. It is true, that some of the abnormal approach much nearer to their natural prototypes, than others; but there is not one, which has not its analogue in the healthy structure. Thus, to use the words of our author, encephaloid bears a striking resemblance to the substance of the brain; melanosis to the coloring matter of the skin (in the negro;) scirrhus to the dermoid texture; and tubercle, at least in some of its forms, to fibro-cartilage. We might extend these comparisons by a reference to the terms mammary, pancreatic, lardaceous, &c. employed by surgeons, to designate varieties of carcinoma, in its earlier stages, when the resemblances of structure are quite as great, as another variety bears to the brain, from which it has received the name encephaloid. Still further, softened tubercle resembles pus, classed it is true with the heterologous products, but presenting so many points of resemblance to mucus, that for ages the profession have exercised themselves on the means of distinguishing, in certain cases, one from the other. The calcareous masses found in the lungs, which in the opinion of Andral have taken the place of tubercles, resemble bone; and some urinary calculi have the same resemblance, while others are but the solidification of matters produced from the kidneys in their healthy condition, and some times found in the circulation.

Those products of morbid secretion, which bear the least resemblance to the tissues into which they are made, are most pernicious, and, at the same time, the most perishable, and vice versa. Thus tubercle, encephaloid and scirrhus, may be contrasted with melanosis, osseous concretions, many encysted tumours, and the false membranes, most of which injure by their mechanical action only; and are not liable to the decomposition, which, sooner or later, awaits those deposits which in their molecular constitution are further removed from the normal tissues. It is during this decomposition, the necessary result of their imperfect organization and vicious or low vital endowment, that the destruction of the tissues which constitute their matrices or beds, takes place; attended with such constitutional derangements, in both the solids and fluids, as very generally prove fatal. Two questions of deep importance to the practical physician are here suggested. First, may we hope to discover how to prevent these deposits? Second, shall we ever acquire the means of protecting the organs and constitution against the destructive consequences of their decomposition—cause them, as it were, to slough away, and the original tissue to be replaced, or a substitute for it formed? We may, indeed, add to these another inquiry—will it be possible to keep these deposits from undergoing decomposition, still suffering them to remain, like encysted bullets, "in the flesh?" We shall not attempt to answer either of these questions.

Having indulged ourselves in these preliminary observations, on some of the first principles of pathological anatomy, we shall proceed to give a more detailed account of the work before us.

In the first chapter, Professor Gross, has presented a general view of the phenomena, causes and nature of inflammation; in which we find much to commend and but little to condemn. From page 23 we make the following extract:

"It has been already intimated, that diseases are functional or organic. As it is of the latter class that we shall more particularly treat in the following pages, it will be proper that we should speak of them somewhat at length. Before proceeding further, however, it behoves me to explain what I comprehend by the term organic. By pathological anatomists, the word is generally employed to denote some permanent change in the textures of an organ; but, in the sense that I would use it, I would not only include under it all such lesions, but also every temporary alteration which the tissues experience when in a state of disease. The term organic will then have a wider latitude; and, as expressing the same thing, we shall often have occasion to use the word structure. If this accetaption be adopted, it may perhaps be doubted whether, under any circumstances, there can, strictly speaking, be a functional disease, or, in other terms, a mere aberration of the physiological state of a part, without some change in its anatomical elements. The question, at all events is not settled.

"Bearing in mind the above definition, it may be assumed, as a general proposition, liable to few exceptions, that all organic diseases, whatever be their seat or extent, are the result of inflammatory action, either of an acute or of a chronic kind. To many, this proposition may be startling; nevertheless, if it be carefully examined, it will be found, I doubt not, to be grounded on fact. The truth of this remark will appear more evident as we proceed."

The first of these paragraphs may present a great truth, but the evidences of it we confesss are not always conspicuous, though, from analogy, we feel almost warranted in the conclusion. The minuteness of the parts in which many morbid actions are seated, must forever render an observation on their appearance, in health or disease, extremely difficult; but we can scarcely conceive it possible, while all the anatomical elements—solid and fluid—continue precisely the same, in their relative quantities and positions, that an altered action can be established in it—we mean an action, that should vary from the natural except in degree. Still, the causes of disease are not necessarily mechanical, but agents which exert their influence on the contractility and sensibility of the tissues, and change their mode of action through the medium of those attributes or properties. Now we may suppose, that simultaneously with that change of action, there is a corresponding alteration of structure—not, it is true, of a *permanent* kind, for it may endure no longer than the morbid action itself, and may consist in nothing more than an increased or diminished quantity of blood in the part—a variation in the relative proportion of arterial and venous blood—and an augmentation or diminution of the interstitial secretion. In addition to these suppositions, we may, however, assume that the molecules of the tissues themselves undergo some change in their relative positions, for the *progress* of diseased action invariably presents us with softening, induration or transformation.

But while we concur with our learned author, in the belief, that altered actions are probably always accompanied by altered organization (embracing in the latter term both the solids and fluids in due proportions,) we are not prepared to assent to his deduction from this proposition, that all diseases are necessarily inflammations; for we can readily conceive that many other modes of altered action and structure, than the inflammatory, *may* exist, and so change the nature of the products of nutrition and secretion, as to present pathological products of a determinate and permanent character. But having already discussed this subject to some extent we shall not enlarge upon it here.

In connexion with these remarks, we give another extract:

"The second proposition that may be stated is, that every inflammation, irritation, or morbid action, is originally of a local nature; that is to say, it makes its impression in the first instance always upon some particular part, texture, or organ. After this inflammation has continued for a longer or shorter period, it often happens that it extends to and implicates other structures. If the mucous membrane of the stomach, for instance, be fretted, the morbid action accruing from this cause will be confined at first to that lining; or, in more comprehensive terms, the disease will be strictly local in its character: by degrees, however, as the disorder progresses, the adjacent parts, such as the submucous cellular tissue, become affected; and, spreading still further, it next invades the muscular fibres of the organ, and, finally, the peritoneal covering. It is in this manner that most affections, which are originally local, extend their sphere of action, so as to become general, whether they be considered simply in reference to one organ, to several, or to a great number of them."

We should prefer the word partial to "local" in the beginning of this extract. The noxious agent which exerts its influence on the cutaneous surface of the body, or the internal parietes of the arterial system, cannot be said to act locally, though its action is but upon a *part* of the body. In his explanation of the extension of morbid actions through the system, our ingenious author does not display his usual perspicuity. He would seem to deduce the affection of *distant* organs, from the same principle or mode of diffusion, by which a morbid action is transmitted from tissue to tissue in one organ. If this were the case, it strikes us, that a distant organ could not be reached, but through morbid action in *all* the intervening parts, as the skin becomes ultimately affected when a suppurative inflammation commencing in the substance of the liver, makes its way to the surface; or a caries becomes the cause of a circumscribed inflammation, progressively set up in all the tissues which lie over that part of the bone which is affected, until the skin is at last involved. These examples of propagation, although regarded by Hunter as sympathies, result, perhaps, from a kind of physical necessity, founded on the immediate anatomical relations of the tissues of the part; and ought not, it strikes us, to be referred to the same principle which causes an inflammation of the liver to excite a pain in the shoulder, or the other great principle, which makes it a fruitful source of morbid sensibility of the nervous system, by the impress on that system of the unexcreted elements of the bile. Our author in other parts of his book gives abundant evidence of a familiar acquaintance with these great principles, and, therefore, perhaps thought a reference to them in this place unnecessary.

"The time of life which seems to be most obnoxious to this disease, (inflammation,) is from the first to the tenth year, nearly one half of the entire mortality occurring during this interval. Affections of the cutaneous, mucous, and lymphatic systems, are particularly rife during this period, and carry off an immense number of children. Scarcely less common is inflammation of the arachnoid membrane. Pleuritis, pneumonitis, cerebritis, and hepatitis, with carditis, phlebitis, and arteritis, are comparatively frequent before the age of manhood; from thence on, however, they are by no means unusual, and prove a fruitful sourceof destruction. Diseases of the genital organs are rarely observed before the age of puberty; anterior to this period, these structures seem, indeed, in a great degree, to lie dormant in the system. Once roused into action, however, they deeply sympathize with the other viscera, and hence the frequency of organic maladies of the uterus, the ovaries, breasts, and testicles, towards the decline of life. Affections of the urinary bladder are comparatively rare in the young, whilst they are very common in the old."

We commend the first part of this comprehensive paragraph to all our practical readers. The frequency of acute inflammatory affections in infancy and childhood, is much greater than many of us suppose.

The following extracts will be read with interest.

"The disease before us does not occur with equal frequency in all the organs and tissues of the body. There are some parts, in fact, in which it has been doubted, though, as I think, without any foundation in truth, whether this affection ever takes place. Such are the nails, the epidermis and the hairs. These structures are supposed, by general anatomists, to be destitute of vessels, and, therefore, incapable of performing any vital action. This however, is merely a conjecture; the fact remains to be proved, and, for my own part, I feel as certain that these textures are susceptible of inflammation, as that the liver is, the stomach, or any other organ.

"The cellular, mucous, serous, and dermoïd textures are particularly prone to inflammation. In children, this is different. In them, the cutaneous and mucous textures are infinitely more liable to inflammation than the cellular tissue and serous membranes—the frequency of their attack being, as nearly as can be, in the order here stated. It is here that the disease can be studied with the greatest advantage, both as it respects its phenomena and modes of termination, inasmuch as it is usually well marked, intense in degree, and rapid in its progress. The synovial membranes, the fibrous envelopes, the bones, ligaments, and cartilages, with the muscles and their tendons, inflame with difficulty; but when the disease has once fastened upon them, they readily yield to its influence, the sufferings are often excessively severe, and the consequences very serious. The blood-vessels, nerves, and absorbents, are all more or less liable to phlegmasia. The conservative powers of these structures, especially of the former, is remarkable, and is strikingly evinced in cases of gangrene, where, as will be shown hereafter, they frequently retain their vitality amidst the half putrefied mass.

"Of the organs, some are more ready to take on inflammation than others. Those which are most frequently affected, at least in this country, are the lungs, spleen, liver, uterus, and brain. The heart, ovaries, thyroïd body, pancreas, prostate gland, testicles, and kidneys, are comparatively rarely the seat of this disease."

The following are our author's views of the structure of the capillary vessels, in all cases the seats of inflammation:

"The walls of the capillaries, as may be imagined, must be extremely thin, delicate and transparent, otherwise it would be much easier to discern them. Bichat states that they are formed entirely out of the inner arterial and venous membrane, the other tunics being excluded, as he alleges, from their composition. An opinion precisely similar to this is advanced by Beclard. He asserts that the parietes of the capillaries are scarcely to be distinguished from the substance of the organs, in which they are situated, and thence draws the inference that they are rather formed out of this substance than that they possess walls of their own, acknowledging, however, at the same time, that it is not impossible that the internal tunic of these vessels is uninterruptedly continued from the arteries to the veins. Admitting, as we have already done, the utter impossibility of determining the precise point at which the arterial tubes in question terminate, and that at which the venous tubes commence, it would seem that the doctrine of the two French anatomists is entirely too exclusive in its bearings to entitle it to our confidence and regard. It is true, neither dissection nor microscopic observation can afford us much aid in solving the difficulty; for the vessels are altogether too minute to enable us to investigate their structure with any degree of accuracy; still, where these means fail us, we are warranted in going to analogy, and in availihg ourselves of its assistance.

"Taking it for granted that the privilege above alluded to properly belongs to us on the present occasion, let us extend our examination to other tubular structures, and see if we cannot find a more philosophical method of disposing of the question than that resorted to by the French anatomists. Let us take the excretory duct of the liver, and follow it along its ultimate ramification in the organ whose secreted fluids it is intended to carry off from the system. In the early part of its course it consists, plainly enough, of two tunics, which, as they extend into the substance of the viscus, become so excessively attenuated that it is quite impossible not only to separate them from each other, but even to distinguish them from the surrounding textures. Now that the inner membrane is prolonged as far as the very point at which the tube terminates, or rather where it takes its origin, no one can for a moment doubt, for the bile is a highly acrid fluid; and hence nature, in order to guard against suffering, has wisely furnished the canal with a mucous lining. But is it reasonable to presume that, because we can no longer discern the external tunic, it must necessarily be wanting? Is it not more philosophical to suppose that both membranes exist, than to say that one is preserved and the other lost? This conclusion involves nothing that is either absurd or improbable; and, although not founded on actual observation, it is much more in conformity with sound anatomy and physiology than any other that has been framed in relation to the subject. If, now, we apply this mode of reasoning to the capillaries, it will at once be perceived that the theory of

Bichat and Beclard is untenable; and that these vessels, instead of possessing, as they imagined, only one tunic, have precisely the same number as the arteries and veins, between which they are situated. If this idea be adopted, it follows, as a necessary corollary, that the capillaries are nourished and animated, like the rest of the vascular system, by vessels still more minute, and by nerves so excessively delicate as to elude even the most powerful microscope."

Prof. Gross' conclusions as to the structure of the parietes of the capillaries have been confirmed by actual observation. In the 877th page of Muller's Elements of Physiology, received in this country since the preparation of the Professor's work, it is stated that by the aid of a high magnifying power, Dr. Schwann has distinguished delicate and indistinct *transverse* fibres in the parietes of the smallest arteries of the mesentery of the frog, and that even the *capillary* vessels of the same part have similar fibres; "a fact," says Muller, "which decides the question, as to capillaries having distinct parietes." Dr. Baly, the London translator, adds that "these *circular* fibres are arranged as in *arteries.* It is necessary to employ a high magnifying power, and rather a feeble light; but they may be seen in the dead, as well as the living frog." In our reasonings, then, upon the influence of agents and on the functions and morbid states of the capillaries, we must be guided analogically by observations made on visible arteries.

Before dismissing this chapter of our author's work, it will be but justice to him and to our readers, to furnish them with an extended extract, that they may judge for themselves of his manner, and this we shall do without comment:

"The very first step in the process of inflammation is an altered sensibility of the part, produced by some hurtful agent, which the system makes an effort to dislodge. To effect this, the local impression is reflected upon the cerebro-spinal axis, and through this again upon the heart, which being sympathetically incited to increased action, more blood flows to the part concerned than it is accustomed to receive, at the same time that the capillaries are perceptibly dilated. Those who maintain that the capillaries possess an inherent contractility, by virtue of which they aid in the circulation, will probably feel disposed to deny the agency of the heart in bringing about this preternatural determination of blood; to such I will only say, that if they will carefully study the subject, they will arrive at a different conclusion. That these vessels do contract and dilate, no one will dispute; for the experiments of Hunter, Wilson Philip, Thompson, Hastings, and other writers, have fully decided this point; all that I contend for is, that the capillaries have no vermicular movement, and therefore they are incapable of carrying on the circulation without the direct influence of the heart. In the inceptive stage of inflammation, this sympathetic action of the heart is no doubt so slight as frequently to escape the attention of the observer: as the disease progresses, it assumes a more distinct character, and can always be easily recognized.

"The phenomena above alluded to, namely, the preternatural influx of blood and the dilatation of the capillaries, can be easily detected by exciting irritation in the mesentery of a rabbit, the tail of a tadpole, the fin of a fish, or the web of a frog's foot, parts which are perfectly transparent, and therefore well calculated for the purpose. On viewing these structures with a microscope, in the sound state, numerous channels will be observed filled with blood, the red globules of which roll along in the most regular and beautiful order. If they be now irritated with spirits of wine, hot water, or diluted acid, the little rivulets just referred to will be found to become dilated, from the manner in which the blood is crowded into them by the heart, which in order to remove the local difficulty, is excited into sympathetic action. In a few minutes hundreds of vessels, which were previously invisible, will be seen shooting out in different directions, and connecting themselves with the sides of those that appeared in the first instance. These are not new channels, but old ones appertaining to the second class of capillaries, which are rendered evident by the intromission of red particles, which are either excluded in the healthy state, or pass along them in so slow and gradual a manner as to elude the eye of the beholder. The little bodies which are thus introduced do not circulate, at first, with the same facility as in the other parts of the body; for, as the dilatation of the little rivulets takes place by degrees, they have to force their way, and hence, after having advanced a short distance, they retreat slightly immediately after each pulsation of the heart, rebounding, as it were, upon each other. In this manner they travel on, surmounting every obstacle, until they finally reach the corresponding capillary veins, into which, as they are considerably more capacious, they rush, as into a vortex. Such are the initial steps of inflammation. If the process be now checked by the removal of the exciting cause, the phenomena referred to gradually disappear, and the part recovers its natural tone and condition.

"If, on the other hand, the inflammation be allowed to proceed, another series of changes may be witnessed, surpassing, if possible, in point of interest, those we have just described. The circulation now completely ceases, the blood assumes a dark modena color, and the coats of the vessels are rendered so soft as to be liable to give way on the slightest force. With these alterations the healthy functions of the part are suspended: it is red, hot, painful, and tumid; and its molecular intervals are filled with serosity or coagulating lymph. In this stage of the malady, the capillaries contain thick, viscid, partially clotted blood, which adheres with great tenacity to their inner surface, and opposes an effectual barrier to artificial injection, or to the removal of the fluid by pressure or ablution. In violent cases the blood escapes from the diseased vessels, and, forcing its way along the cellular tissue, forms new channels, through which it afterwards continues to circulate. This interesting phenomenon, which has been frequently noticed by Kaltenbrunner in the inflamed mesentery of the rabbit, is strictly analogous to what occurs in the organization of adventitious membranes,—a subject to which the attention of the reader will be subsequently directed.

"Inflammation, it will thus be seen, is a gradual process, which is preceded and accompanied by certain stages. Of these, three are recognized by Kaltenbrunner. The first he denominates the stage of incubation; the second, the stage of congestion; the third, the stage of inflammation, properly so called. Each of these is characterized by particular phenomena, the most important of which have been already described, in the order, as nearly as may be, in which they appear. To this arrangement I can see no special objection: it should be recollected, however, that it is altogether artificial, and that the stages which it recognizes are frequently so blended as to render it impossible to distinguish them from one another. Contrary to what might be inferred from analogy, Kaltenbrunner has ascertained the singular fact, that more time is usually required for inflammation to be developed in highly vascular organs, as the lungs and peritonæum, than in parts in which the circulation is more tardy and less perfect, as the liver and kidney. It is worthy of remark, however, that when the disease is once fairly established, it progresses much more rapidly in the former than in the latter of these structures.

"Another striking phenomenon is the distended condition of the larger vessels leading to the inflamed part. When the disease is at its height, the congestion often extends to a considerable distance; the blood is unnaturally dark, thick, and viscid, and artificial injection is difficult, sometimes impracticable. It has been alleged that the larger arteries in the immediate neighborhood of the lesion occasionally pulsate with preternatural force and frequency; but this is an assertion which is unsupported by proof, and which is, moreover, in direct opposition with every principle of physiology. The intensity of the morbid action is generally greatest at the centre of the inflamed part, from which it is gradually, and, in some instances, suddenly diminished, until it loses itself in the circumjacent textures."

Chapter II treats of the effusions of serum. These are among the earliest and most constant of the effects of inflammation. Indeed, we may presume, that in all cases of inflammation, whatever other matters may at length or ultimately be poured out, serum more or less changed in its constitution, is the first secretion. Being a proximate effect of the vascular turgescence which it no doubt operates to diminish, we may regard its secretion, as one of the modes in wbich the cure by *resolution* is effected. At least many such cases must of necessity be confounded with those in which the inflammation is said to be discussed. When seated in certain parts of the body, this mode of termination is harmless, as the effused fluid may not much derange the functions of the organ, and will be soon absorbed; but in others, as the pericardium and ventricles of the brain, its presence is of serious import.

Under this head professor Gross has sought for aid to his favorite hypothesis, that all morbid secretions are the effect of inflammation. We shall let him speak for himself.

"It has been already hinted that serous effusion is the result of inflammation, usually of a very mild grade. That this is true, as a general rule, very few will attempt to dispute; the exceptions, if there be any, are certainly very rare, and have not hitherto been satisfactorily pointed out. A few facts, clearly and concisely stated, will assist in determining this problem.

"It has been alleged, in the first place, that serum is occasionally effused when there is an obstacle simply in the circulation, without any concomitant inflammatory action. It is a matter of common observation with the physician, that anasarca of the lower extremities often arises from obliteration of the femoral, external iliac, and ascending hollow vein; and the face, neck, and arms are frequently loaded with serum from compression of the vessels whose duty it is to return the blood to the right side of the heart. When the portal vein, or any one of its principal branches, is obstructed, abdominal dropsy, or ascites, follows. Contraction of the right auriculo-ventricular orifice, or disease of the valves of the pulmonary artery, impeding the passage of the blood, and compelling it to regurgitate into the inferior cava, produces the same result, together with œdema of the legs and feet. These examples will be sufficient for the subject which they are intended to illustrate. Let us now endeavor to ascertain how far they are dependent upon inflammation, or whether they are the result merely of mechanical obstruction? It is frequently extremely difficult to ascertain the condition of the seat of the effusion by anatomical inspection. In ascites how often does it not happen that there is the most copious accumulation of water, caused obviously by inflammation! and yet, on examination after death, there is scarcely a well-marked trace of the latter malady. That there are cases, then, of serous effusions, in which the ordinary phenomena of phlegmasia, particularly the discoloration, entirely vanish on the approach of death, or during the last struggles of life, cannot be doubted; indeed, it is not improbable, I think, that there are instances in which this disposition occurs a long time before the individual expires. The absence of redness, therefore, does not prove that there was no inflammation; for the existence of this lesion is sufficiently evinced by the presence of the watery accumulation, and the opacity of the affected membrane. Should there be, in addition, specks, patches, or bands of fibrin, all doubt on the subject must vanish.

"Such, then, being the difficulty of recognizing the presence of inflammation, where every symptom during life gives indubitable evidence of its existence, can it be wondered at that, in the instances above referred to, pathologists should still consider the effusion of serum as the result merely of mechanical obstruction? The question may now be asked,—can such an obstruction exist, to any considerable extent, without producing a state of parts analogous to, if not really identical with, inflammation? I would answer, no. Let it be supposed that the obstacle exists in the ascending hollow vein. This vessel is destined to return the blood from the inferior extremities, the pelvis and abdomen, to the right side of the heart. But, failing in the accomplishment of this object, from the difficulty adverted to, the blood is interrupted in its passage upwards, and congestion of all the vessels, both large and small, is the result. This congestion is not transient, but permanent; and it is scarcely reasonable to presume, judging from our knowledge of the circulation, that this state could exist long without producing an altered condition of the sensibility of the parts affected, attended with more or less redness, and effusion of serosity. The peritonæum and cellular tissue of the limbs are the structures which receive the brunt of the difficulty, and these are parts, it is well known, which are most liberally supplied with serous capillaries. But, it may be said that the effusion may result from perverted action, from irritation, or disturbed function: all this may be true, and yet not in the least invalidate our position. Every body knows that in inflammation there is perverted action, or deranged function, with irritation, or altered sensibility. These terms, therefore, if they mean any thing at all, only denote certain conditions—not the cause of these conditions; as redness, heat, pain, and turgescence are not inflammation, but only so many symptoms of it.

"The preceding remarks are equally applicable to the watery effusions of the serous textures, which occur in association with organic diseases of the glandular and parenchymatous viscera. A large scirrhous tumor of the liver, seated so superficially as to encroach upon and fret its serous investment, is often attended with ascites, although the portal circulation is in no wise obstructed or embarrassed. In the same manner hydrothorax is sometimes induced by tubercles of the lungs; hydrocele by carcinoma of the testicle; hydrocephalus by heterologous growths of the brain. In all these instances the effusion of water is the result, unquestionably, of inflammation, lighted up in the serous covering of the respective organs, by the morbid deposit acting in the capacity of a foreign substance. The dropsical accumulations which supervene upon scarlet fever, measles, and other eruptive diseases, can be traced, in most cases, directly to phlegmasial irritation of the serous membranes.

"Taking into consideration the preceding facts, and the reasoning founded upon them, the conclusion is obvious that the effusion of serosity, no matter in what part, organ, or region it occurs, is the result, *invariably*, of a process analagous to, if not strictly identical with, inflammation. This process, we repeat, is often very imperfectly marked, both during life and after death, so that the ordinary phenomena of phlegmasia are in no way manifest to our senses. That this conclusion is fully borne out by the premises, is, I think, sufficiently evident; and we shall, therefore, dismiss the subject, in the hope that what has been stated will have a tendency, at least, to arouse the attention of the profession to further investigation respecting it."

On this able and interesting extract, we shall make but one or two passing remarks. Our author insists, that the various effusions to which it refers, are the "result *invariably* of a process *analagous* to, if not identical with inflammation." With this modification of his doctrine we are content to receive it. The analogy may extend as far as congestion and increased serous secretion, are concerned; but congestion is not inflammation. It is, moreover, by no means certain, that all cases of increased serous effusion, are preceded by congestion, for the secretion may itself prevent, seeing that it can cure congestion. It remains to be shown, that we cannot have increased serous secretion without congestion. A secreting surface when it pours out more than its usual quantity, doubtless receives more than its usual portion of blood, in a given time; but it does not follow, that the capillaries become engorged.

The third chapter treats of effusions of coagulable lymph, to which our author has with propriety applied the term *lymphization.* These are the sources of nearly all the analogous formations, and their study is of the deepest interest to the surgeon and physician. Coagulable lymph, we regard with our author, as exclusively the product of inflammation.

"With the appearance of this substance, every one is familiar. In the majority of instances, it is of a light opaline cast; in others, it is of a pale color, cineritious, of a milky white, or reddish, from the admixture of hœmatorine. When first deposited, it is soft, fluid, and somewhat ropy, allowing itself to be drawn out into little filaments: after a while, however, as its watery particles are being removed, it assumes greater consistence, and is finally converted, in many cases, into firm, dense structure, having all the attributes of the cellular tissue, fibrous membrane, or even cartilage and bone. The period required for these transformations varies from a few weeks to as many months."

The coagulable lymph must not be confounded with the fibrin of the blood, which is but one of its elements—howbeit the most important—for the albumen of the serum enters likewise into its composition. It is, indeed, a secretion not merely in the sense of something extravasated from the vessels, but in the more legitimate sense, of something formed out of the blood and discharged from the capillaries. To its production the red globules *may* also contribute, and, perhaps, to their presence, altered it is true, and made to harmonize with the other altered elements of that fluid, may be ascribed the spontaneous or independent formation of blood in the effused lymph.

Fibrin, as constituting a false tissue or formation, is found in certain cases of sanguineous extravasations into the cellular membrane or upon the serous surfaces, or when the currents of circulation have been arrested in sections of vessels. Under such circumstances, the fibrin coagulates, the coloring matter is decomposed, and the serum absorbed. An adhesion takes place between the coagulated fibrin and the adjoining surfaces, and, finally, the former receives vessels from the latter. This adhesion is brought about, according to our author, by inflammation, excited in the normal surfaces, by the irritation of coagulum; but Carswell and other pathologists, regard it, and we believe justly, as identical with the adhesion of cardiac polypi to the columnæ carneæ, a mode in which it is probable vegetations of the valves of the heart are formed. Thus the distinguished anatomist whom we have just mentioned, has established two genera of analagous formations—one from coagulable lymph, the other from fibrin; but our author admits only the latter; or, to speak more accurately, regards the concurrence of the former, as essential to the union of the latter with the living textures.

The analagous tissues which our author admits, are the cellular—serous—mucous—cutaneous—vascular, including the erectile—adipous—horny, including the cuticle, hair and nails—fibrous—cartilaginous—cartilaginous and osseous; each of which will be considered in connexion with its archetype in the healthy system.

Lymphization is nature's standing prescription for a great variety of lesions. By it incised wounds are immediately healed, cut arteries closed, and lacerated wounds, attended necessarily with sloughing, filled up; broken bones are reunited, and necrosed bones replaced; the diffusion of pus restrained; tubercular cavities occasionally walled; the fatal effects of intestinal mortification and sloughing averted; aneurismal arteries obliterated; great surgical operations rendered admissible, and the stumps of amputated limbs speedily healed! On the well-known but absurd aversion of French surgery to the last, our author has the following merited criticism:

"Much good has also resulted in respect to the treatment of incised wounds, whether produced by accident, by the removal of a tumor, or the amputation of a limb. In all these instances it is customary, in every part of the civilized world, with the exception, perhaps, of France, to endeaver, if possible, to bring about union by the first intention; the surgeon well knowing that if this can be effected he will save himself much trouble, and the patient no little suffering and inconvenience. When we reflect upon this subject, it is extremely difficult to account for the great apathy and silly hesitancy which the Parisian practitioners exhibit, with regard to this plan of treatment, about the propriety of which there ought to be but one opinion. In this respect they certainly are fifty years behind the present state of the science,—a circumstance which is so much the more surprising, when it is recollected that they have decidedly the best schools of pathology in the world."

But good and evil are associated, and while lymphization brings us so much of the former, it imposes not a little of the latter. The adhesive and plastic character of lymph, and its long continued contraction or shrinking up, are the sources of these difficulties. Thus, after an extensive loss of skin by sloughing from fire, the new textures often produce deformity, and sometimes an inconvenience which no surgical operation can obviate; again in inflammations of the cheek, adhesions and indurations compress the jaws against each other; from the same cause, the eye-lid becomes agglutinated to the ball; the larynx fatally obstructed with false membrane; the urethra nearly closed up; the intestines combined into a great bundle; the lungs and pleura cemented to each other; and the solid viscera compressed till they fall into atrophy.

What then can be of deeper interest to the physician and surgeon, than the study of lymphization! How important to know the means of promoting it when necessary, and of averting it when and where it would be mischievous!

Our author next treats of suppuration. He adopts and enforces the sound doctrine that *the formation of pus in whatever part of the body occurring, is the result of inflammatory action, either acute or chronic, simple or specific.* Some of the large and cold abscesses are the offspring of very chronic inflammation—others no doubt are but collections of pus secreted in an inflamed part, absorbed and subsequently deposited in another. Pus may be formed without any solution of continuity. This happens especially in the lungs and their investing membranes. Purulent expectoration without ulceration is not very uncommon, and empyema is a collection of pus in the cavity of the pleura, not, we presume, the consequence of ulceration. We have met with two cases in which the quantity was so great, as to indicate secretion from an extensive surface, and the recovery after paracentisis so rapid and perfect, as almost to demonstrate, that the pleura could not have been the seat of ulcerative absorption, or the subserous tissue the bed of an abscess.

"Pus, when first effused, generally appears in the form of distinct globules, which are dispersed through the affected structure, and can be easily recognized by their pale yellowish color. As the purulent particles increase in number, they gradually become confluent by the absorption of the part concerned, and in this way the matter is at length collected into an abscess."

The account here given varies somewhat from the reports of several other observers. Autenrieth affirms, that if some of the watery moisture, which exudes from the surface of an inflamed part after the pus has been removed, is collected between two transparent thin plates of talc and allowed to lie in the wound, globules are seen to form gradually in it, to enlarge and become opaqe, while if the fluid is removed altogether from the atmosphere of the living textures, no such change takes place in it. Brugman, also, states that if a supurating surface has been washed clean, the pus is seen to be secreted as a clean fluid, which afterwards become thick and opaque.—Muller Elem. Phys. 469.

The experiments of Gendrin have shown, that pus is formed out of *all* the elements of the blood. He has seen that fluid when extravasated gradually transformed into pus. To this end, however, it must be lodged in the midst of an inflamed tissue.

An attentive study of the characters of pus is essential to the surgeon. These characters will sometimes indicate to him the tissue affected, and very commonly the state of the secreting surface—sometimes even that of the constitution.

Pus is very often imbued with a specific quality—syphilitic, variolous, gonorrhœal, &c., which does not seem to modify its sensible properties. It would be interesting to ascertain, whether the pus which might be secreted under an accidental solution of continuity, in the vicinity of a chancre, or in the midst of variolous pustules would possess a specific character.

But we must turn aside, and bring this extended, perhaps tedious review to a close. There remain many other subjects to be discussed, which we shall make the topics of other articles. Having completed the division of *general* pathological anatomy, we shall from time to time indulge ourselves in the presentation of some of the leading facts touching the morbid anatomy of *particular* organs, connecting them with pathological speculations, and occasional therapeutic remarks.

The high estimate we place on Professor Gross' work may be inferred from the copious presentation we have made of a part of its contents in this article, not less than the intimation we have just given in regard to what remains. If we are not greatly mistaken, its publication will give to the study of pathological anatomy in the United States, and especially in the west and south, where the author is extensively and advantageously known, a decided impulse. The work bears intrinsic evidence of varied and patient research; and while the author has availed himself of nearly all the standard works of Europe, he has not neglected to bring forward the rich results of his own experience. For some time called upon by a large proportion of his brethren in the city where he lives, to make *post mortem* inspections for them, we know his opportunities to have been greatly multiplied; and have often had occasion to mark his accuracy and minuteness. For *our* surgeons and physicians, we regard his work as decidedly superior to that of Andral. That distinguished pathologist, it is well known, reserved his practical remarks for his *Clinique Medicale*, scarcely ever met with in this country; but Professor Gross has sought to connect his with the different pathological appearances which it has been his duty to describe. Moreover, he has given us the morbid anatomy produced by our *own* diseases; and an unusually large proportion of his cases have been in private instead of hospital practice, all of which is calculated to give his facts a special application to western practice.

